# Retraction: Multiwalled carbon nanotube wrapped nanoflake graphene composites for sensitive biosensing of leviteracetum

**DOI:** 10.1039/c9ra90046b

**Published:** 2019-06-19

**Authors:** Jagriti Narang

**Affiliations:** Amity Institute of Nanotechnology, AMITY University Noida India jags_biotech@yahoo.co.in +9811792572

## Abstract

Retraction of ‘Multiwalled carbon nanotube wrapped nanoflake graphene composites for sensitive biosensing of leviteracetum’ by Jagriti Narang *et al.*, *RSC Adv.*, 2015, **5**, 13462–13469.

The Royal Society of Chemistry has been contacted by the President of the Amity Science Technology & Innovation Foundation, Amity University Uttar Pradesh regarding an investigation into the authorship of this *RSC Advances* paper and concerns about discrepancies in the data and results presented in the article.

(1) The investigation by Amity University Uttar Pradesh has concluded that Nitesh Malhotra and Nidhi Chauhan should not have been included as co-authors because they only made a minimal contribution to the study. Their contributions were that Nitesh Malhotra provided the leviteracetum drug and Nidhi Chauhan provided the multiwalled carbon nanotubes.

Aside from the institutional investigation, the corresponding author has also confirmed to us that C. S. Pundir should not have been included as a co-author as their contribution was to check the language in the manuscript.

The contributions of Nitesh Malhotra, Nidhi Chauhan and C. S. Pundir should have been acknowledged in the article, but did not justify authorship.

The corrected authorship list and affiliation for this paper are as follows:

Jagriti Narang*^a^

^a^Amity Institute of Nanotechnology, AMITY University, Noida, India. E-mail: jags_biotech@yahoo.co.in; Tel: +9811792572.

(2) I, Jagriti Narang, the corresponding author hereby wholly retract this *RSC Advances* article due to concerns with the reliability of the data in the published article.

The TEM images in Fig. 1a and SEM images in Fig. 2b and c each contain repetitions of a number of distinct shapes within the images.

The XRD data in Fig. 1b contains repetitions of the same peaks within the traces. The peak at 11° in the GNF trace has been duplicated in the MWCNT/GNF trace at 11 and 24° and in the MWCNT trace at 24°. In addition, the peak at 45° in the MWCNT trace has been duplicated at 78° in the MWCNT trace and at 70° and 78° in the FTO trace.

The investigation by Amity University Uttar Pradesh has recommended that the paper should be retracted.

Signed: Jagriti Narang.

Retraction endorsed by Andrew Shore, Executive Editor, *RSC Advances*.

## Supplementary Material

